# Immune checkpoint inhibitors induce acute interstitial nephritis in mice with increased urinary MCP1 and PD-1 glomerular expression

**DOI:** 10.1186/s12967-024-05177-9

**Published:** 2024-05-03

**Authors:** Laura Martinez Valenzuela, Francisco Gómez-Preciado, Jordi Guiteras, Paula Antón Pampols, Montserrat Gomà, Xavier Fulladosa, Josep Maria Cruzado, Joan Torras, Juliana Draibe

**Affiliations:** 1 Nephrology Department, Bellvitge University Hospital. Bellvitge Biomedical Research Institute (IDIBELL), Hospitalet de Llobregat, Feixa Llarga S/N, Barcelona, 08907 Spain; 2 Pathology Department, Bellvitge University Hospital, Hospitalet de Llobregat, Barcelona, 08907 Spain; 3 Faculty of Medicine, Bellvitge Campus, University of Barcelona, L’Hospitalet de Llobregat, Barcelona, 08907 Spain; 4 Experimental Nephrology Laboratory, Institut d’Investigació Biomèdica de Bellvitge (IDIBELL), L’Hospitalet de Llobregat, Barcelona, 08907 Spain; 5 Fundació Bosch i Gimpera, University of Barcelona, Barcelona, 08028 Spain

**Keywords:** Acute interstitial nephritis, Animal model, Immune checkpoint inhibitors, MCP1, Urinary biomarkers

## Abstract

**Introduction:**

Immune checkpoint inhibitors (ICIs) induce acute interstitial nephritis (AIN) in 2–5% of patients, with a clearly higher incidence when they are combined with platinum derivatives. Unfortunately, suitable disease models and non-invasive biomarkers are lacking. To fill this gap in our understanding, we investigated the renal effects of cisplatin and anti-PD-L1 antibodies in mice, assessing PD-1 renal expression and cytokine levels in mice with AIN, and then we compared these findings with those in AIN-diagnosed cancer patients.

**Methods:**

Twenty C57BL6J mice received 200 µg of anti-PD-L1 antibody and 5 mg/kg cisplatin intraperitoneally and were compared with those receiving cisplatin (*n* = 6), anti-PD-L1 (*n* = 7), or saline (*n* = 6). After 7 days, the mice were euthanized. Serum and urinary concentrations of TNFα, CXCL10, IL-6, and MCP-1 were measured by Luminex. The kidney sections were stained to determine PD-1 tissue expression. Thirty-nine cancer patients with AKI were enrolled (AIN *n* = 33, acute tubular necrosis (ATN) *n* = 6), urine MCP-1 (uMCP-1) was measured, and kidney sections were stained to assess PD-1 expression.

**Results:**

Cisplatin and anti PD-L1 treatment led to 40% AIN development (*p* = 0.03) in mice, accompanied by elevated serum creatinine and uMCP1. AIN-diagnosed cancer patients also had higher uMCP1 levels than ATN-diagnosed patients, confirming our previous findings. Mice with AIN exhibited interstitial PD-1 staining and stronger glomerular PD-1 expression, especially with combination treatment. Conversely, human AIN patients only showed interstitial PD-1 positivity.

**Conclusions:**

Only mice receiving cisplatin and anti-PDL1 concomitantly developed AIN, accompanied with a more severe kidney injury. AIN induced by this drug combination was linked to elevated uMCP1, consistently with human AIN, suggesting that uMCP1 can be potentially used as an AIN biomarker.

**Supplementary Information:**

The online version contains supplementary material available at 10.1186/s12967-024-05177-9.

## Introduction

Immune checkpoint inhibitors (ICI) have improved the prognosis of a number of malignancies. ICI inhibit the programmed cell death protein 1 (PD-1)/ programmed cell death ligand 1 (PD-L1)/ programmed cell death ligand 2 (PD-L2) immunoregulatory pathway expressed by immune cells among others, thus enhancing natural anticancer immunosurveillance. Unfortunately, the use of ICI increases the risk of immune-related adverse effects (irAEs). The most frequent renal irAE is acute interstitial nephritis (AIN), which consists of a tubulointerstitial lymphocytic infiltrate leading to acute kidney injury (AKI). The treatment consists of the withdrawal of ICI and the administration of corticosteroids. The exact pathomechanism of AIN remains speculative, including hypersensitivity responses to the drug itself or to previously tolerated drugs and renal antigens.

Renal tubular epithelial cells express PD-L1 and act as non-professional antigen presenting cells [1], when inflammation is present. Cassol et al. observed increased tubular PD-L1 in patients with ICI-related AIN compared to other causes of AIN, but they did not study glomerular PD-1 expression [2]. Pippin et al. reported higher PD-1 expression in glomeruli from aged mice, particularly localized in the podocytes, which was associated with a deterioration in kidney function [3].

ICIs are frequently combined with cisplatin to enhance their antineoplastic effects [4–6]. Unfortunately, recent review in literature has described a higher prevalence of grade 3–5 renal adverse effects in patients treated with this combination [7]. Cisplatin causes proximal tubular renal cells injury by different mechanisms, including inflammation [8]. Several authors have described the release of interleukin (IL) 1β, IL6, Monocyte chemoattractant protein-1 (MCP1) and tumor necrosis factor alpha (TNFα), and the blockade of some of these cytokines is associated with attenuated phenotypes of cisplatin-induced AKI in murine models [9].

The mouse model used to study cisplatin-induced AKI consists of the intraperitoneal administration of 10-30 mg/kg of cisplatin in a single dose and elicits severe kidney injury with high mortality. To date, a preclinical model of AIN associated to immune checkpoint blockade is lacking. PD-1 knockout mice develop a lupus-like glomerulonephritis [10]. There are few reports of the renal effects of the combination of cisplatin and ICI in preclinical models. Spielbauer et al. found a mild increase in serum creatinine in CBA/CaJ mice treated with cisplatin plus anti-PD1 antibodies compared to monotherapy without there being differences in renal histology [11]. Tran et al. did not find differences regarding the renal histology in C57BL6 mice treated with cisplatin combined with anti PD-1 or PD-L1 antibodies compared to each treatment used in monotherapy. However, they did not measure serum creatinine [12].

We hypothesized that the combined administration of cisplatin and ICI might induce acute AIN in mice. Our aim was to comprehensively describe the renal effects of the concurrent administration of both drugs, including its effects on kidney function, urinalysis, levels of proinflammatory cytokines, renal histological injury, and PD-1 expression. Additionally, we aimed at comparing the findings in mice to the findings in a cohort of cancer patients treated with these drugs and diagnosed with AIN or acute tubular necrosis (ATN).

## Materials and methods

### Induction of acute interstitial nephritis in mice

Male and female C57BL/6J mice between 8 and 10 weeks of age from Charles River Laboratories (Willmington, MA) were kept at the Animal Facility on the University of Barcelona Campus Bellvitge at constant temperature, in 12 h dark/12 hour light cycle, with tap water and a standard diet *ad libitum*.

**Fig. 1:**
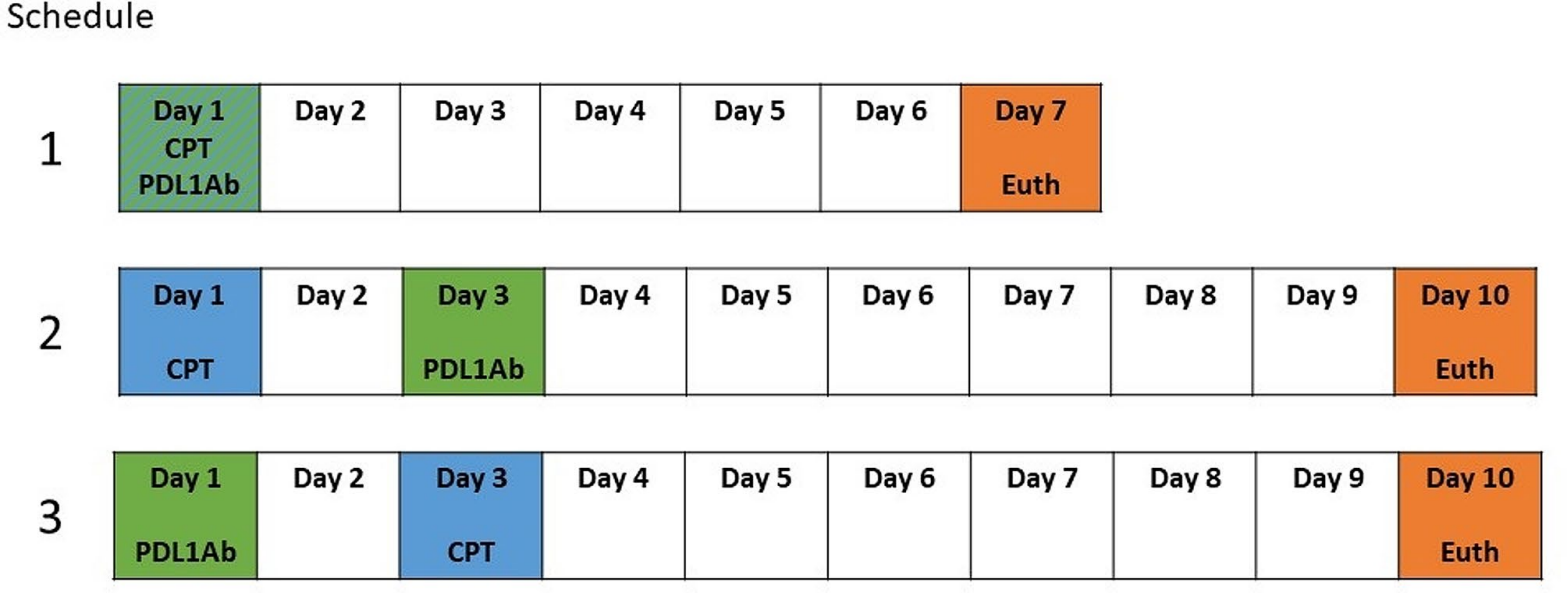
Summarizes the 3 schedules of treatment administered to the mice receiving a combination of cisplatin plus anti-PD-L1 antibodies. CPT- Cisplatin; PDLAb- anti-PD-L1 antibodies; Euth- euthanasia

Mice were randomized so as to receive 5 mg/kg cisplatin (Selleckchem Chemicals, Catalog #S1166) intraperitoneally (ip), 200 µg anti-PD-L1 antibodies ip (InVivoMAb anti-mouse PD-L1 Clone 10 F.9G2, BioXCell [13]) or their combination (randomized into 3 different schedules illustrated in Fig. 1). The distribution of the groups and the treatment schedules are shown in table 1. At the time of euthanasia, blood was obtained via submandibular vein puncture, and urine was collected through direct bladder puncture. Kidneys were obtained and processed for histological studies.

**Table 1:** Distribution of mice among the different treatment groups

Treatment	n
Cisplatin monotherapy	6
Anti PD-L1 antibodies monotherapy	7
Cisplatin + Anti PD-L1 antibodies	
*Schedule 1*	6
*Schedule 2*	7
*Schedule 3*	7
Saline	6

Serum creatinine was determined by an automated biochemistry analyzer. A semi-quantitative assessment of hematuria, proteinuria and leukocyturia was performed using a urine dipstick.

### Mouse conventional histology and immunofluorescence studies

Kidneys were cut longitudinally, fixed in 4% paraformaldehyde and embedded in paraffin. 5 μm sections were stained with hematoxylin and eosin and were examined under a light microscope.

ATN was defined as the presence of hydropic degeneration and cytoplasmic vacuolization of the kidney tubule cells, loss of brush border, desquamation, dilation or tubular hyaline/proteinaceous casts [14]. The presence and grade of ATN was evaluated blindly and graded into (0) absent, (1) mild, (2) moderate, and (3) severe.

AIN was defined as the presence of 2 or more 20x magnification field extension areas of interstitial edema and leukocyte infiltration [15]. The number of areas containing these lesions were counted.

For PD-1 staining, 5 μm sections were dewaxed and rehydrated using an autostainer. Antigen retrieval was performed with a pH 9 citrate buffer. Sections were incubated in Block Buffer (TBS1x + 0.5%triton + 3% donkey serum) for 1 h at room temperature (RT). Afterwards, they were incubated overnight at 4ºC plus 2 h at RT with primary antibody (Goat Anti-Mouse PD-1 Antibody (R&D, Catalog #AF1021)) diluted to 1:10. Thereafter sections were incubated for 2 h at 37ºC with secondary antibody (anti-mouse IgG-cy3) diluted 1:200 and counterstained with DAPI. The slides were stored flat at 4ºC, and images were acquired after a maximum of 7 days using an Axio Imager M2 microscope (Zeiss).

Four random cortical regions of interest (900 μm diameter each) were selected in each kidney preparation. The intensity of glomerular PD-1 staining was scored semi quantitatively (0: no staining, 1: weak staining, 2: moderate staining, 3: strong staining).

### Clinical recruitment and assessment

We recruited all consecutive cancer patients treated with ICI and/or platin derivatives diagnosed with AIN or ATN in our institution between January 2017 and June 2023. Exclusion criteria were: active urinary tract infection, sepsis, and other autoimmune conditions.

The main demographic variables of interest and prior medical conditions were recorded at baseline. Central laboratory tests at baseline were registered.

### Human conventional histology and immunofluorescence studies

The human kidney biopsies were processed similarly to the mice biopsies. Histological grading of the AIN was performed in non-scarred cortical areas and graded depending on the extent: grade 0 (affecting < 25%), grade 1 (affecting 25–50%), grade 2 (affecting 50–75%) and grade 3 (affecting > 75%). The presence of ATN was also registered.

For PD-1 immunostaining, 2.5 μm paraffin-embedded kidney sections were dewaxed and rehydrated. Tissue was incubated with mouse anti-human PD-1 antibody (ab52587, Abcam) diluted 1:500. Thereafter, sections were incubated with a conjugated secondary antibody, and binding was revealed using 3,3-diaminobenzidine as a chromogen with avidin-biotin-peroxidase complex (EnVision Flex K8002 visualization System). The presence of PD-1 staining was semi-quantitatively assessed at 400x.

### Cytokine measurement in mice and human samples

Urine and blood samples from mice were collected at euthanasia. Urine from patients diagnosed with ATN and AIN was collected at the moment of the diagnosis, centrifuged at 2000 rpm and stored at -80ºC. For mice samples, we customized a Multiplex Immunoassay commercial kit in the Luminex platform (Invitrogen ProcartaPlex, Thermofisher Scientific) to detect IL-6, interferon gamma (IFNγ)-induced protein 10 (IP-10)/C-X-C motif chemokine ligand (CXCL)-10, TNFα, and MCP1. For human urine, we tested the concentration of MCP-1 using the same platform and provider in accordance with the manufacturer’s instructions. A Luminex MAGPIX®reader was used.

### Statistical analysis

Data was analyzed using the GraphPad Prism version 8.00 (GraphPad Software), IBM SPSS Statistics Version 22.0 (IBM corp.), and RAWGraphs (DensityDesign Research Lab). To determine the Gaussian distribution of the variables, Kolmogorov–Smirnov was applied. ANOVA test, Student’s t-test or Mann–Whitney U were used to compare the means of quantitative variables, and Chi-square to compare the frequency of qualitative variables. Correlations were assessed using the Pearson correlation coefficient. ROC curves were plotted to evaluate the discriminatory performance of a variable. *p*-Values < 0.05 were considered statistically significant.

## Results

### Administration of cisplatin plus anti-PD-L1 antibodies leads to AKI and induces AIN in mice

**Fig. 2:**
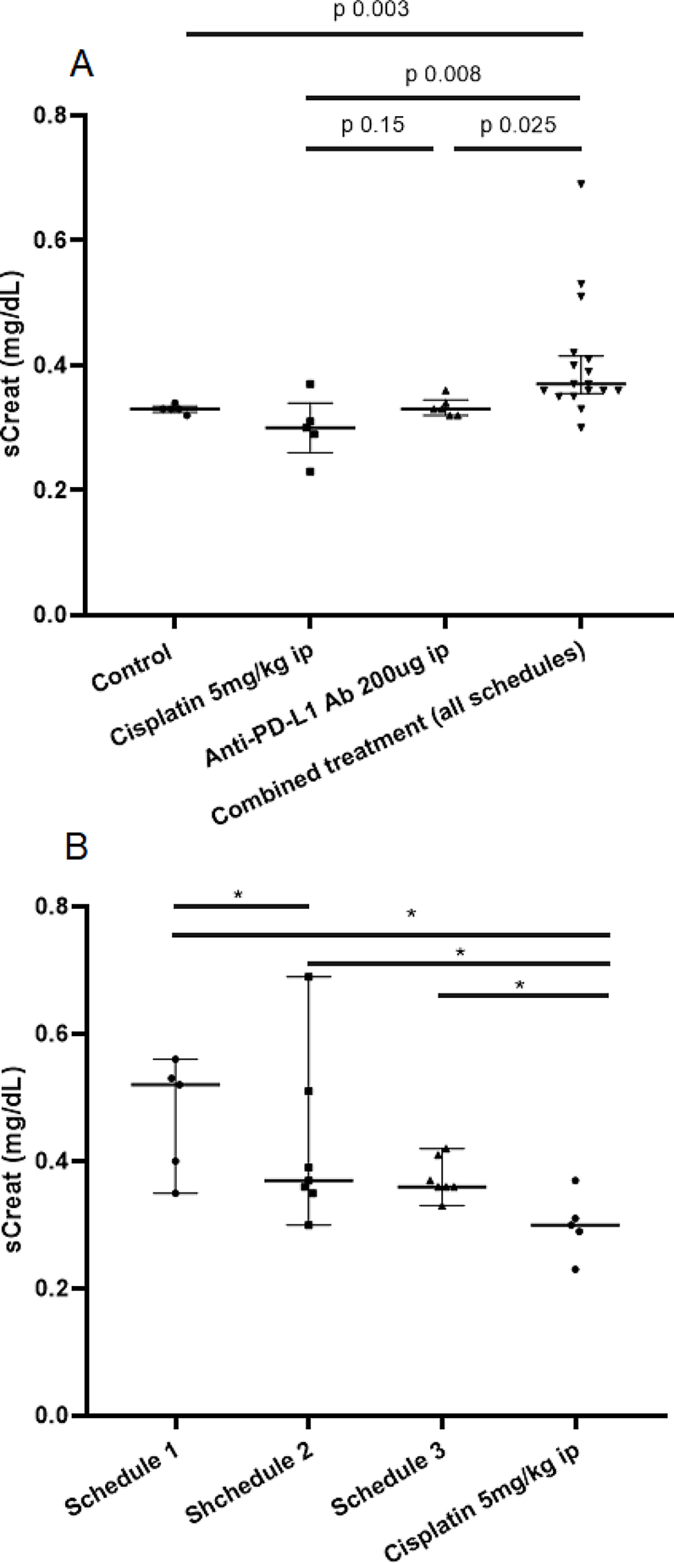
Serum creatinine value in the different treatment groups. (**A**) Serum creatinine depending on the received drugs. (**B**) Serum creatinine in animals receiving cisplatin plus anti PD-L1 antibodies depending on the treatment Schedule. * *p* value of the comparison < 0.05

Animals treated with all combinations of cisplatin plus anti PD-L1 antibodies showed significantly higher serum creatinine compared to animals receiving monotherapy and controls (see Fig. 2). Serum creatinine was higher in mice with AIN (0.49 ± 0.1 mg/dL vs. 0.34 ± 0.06 mg/dL, *p* < 0.0001). There were no differences in the urinary parameters evaluated by dipstick according to the treatment groups (see Supplementary Fig. 1).

AIN was only present in mice treated with cisplatin plus anti PD-L1 antibodies, compared to none being found in the monotherapy groups (p 0.03). We did not find differences in the prevalence of AIN in the different schedules of administration of cisplatin plus anti PD-L1 (data not shown). Moreover, the number of areas of interstitial infiltrates within the kidney section correlated with serum creatinine (r 0.53, p 0.003).

**Fig. 3:**
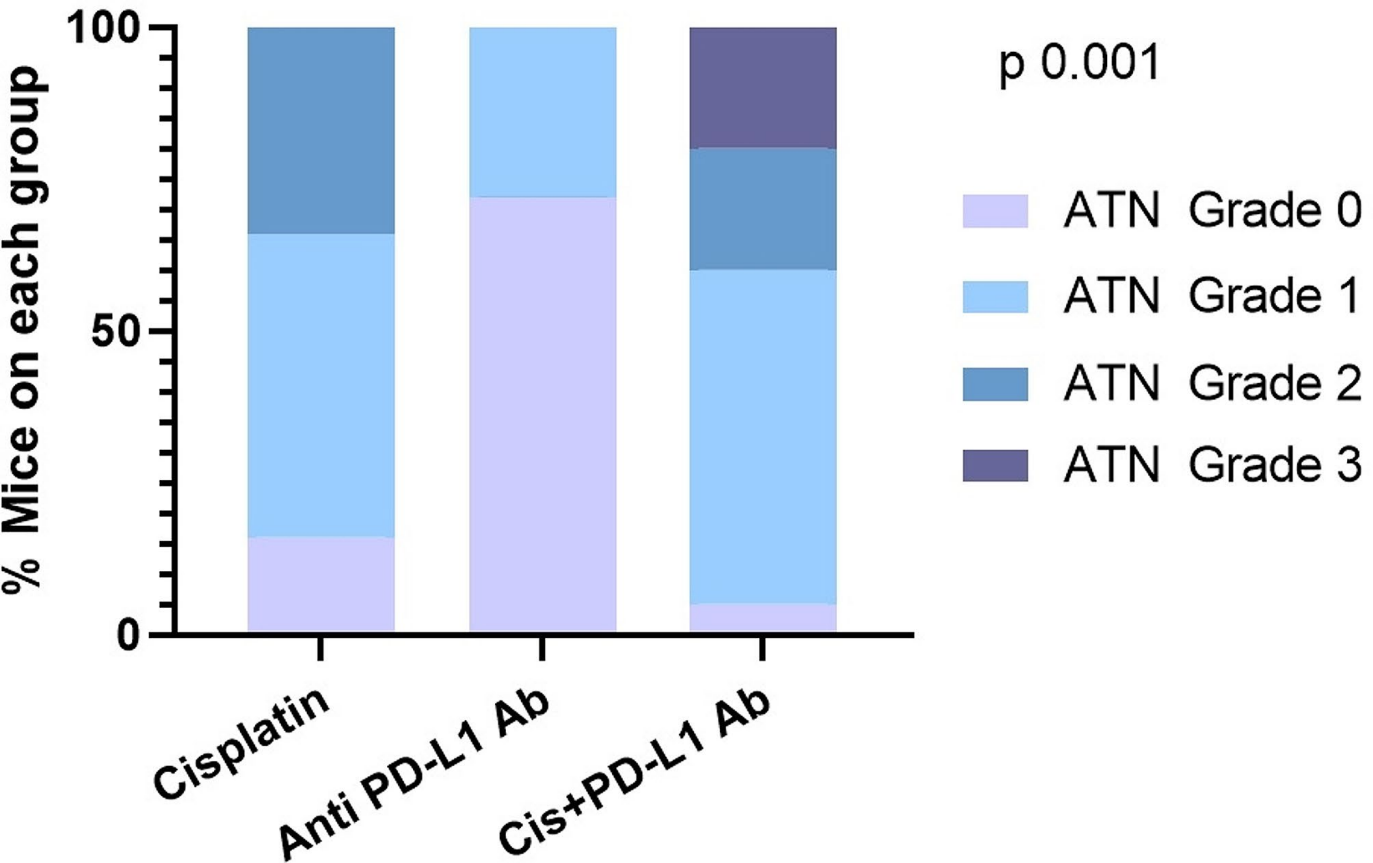
Distribution of the prevalence and grade of acute tubular necrosis among the three treatmEnt groups. Cis– Cisplatin, ATN– acute tubular necrosis

ATN was noticed in all groups. The highest percentage of mice presenting ATN was observed in the group treated with cisplatin plus anti-PD-L1 antibodies (95%) (p 0.001) (See Fig. 3).

**Fig. 4:**
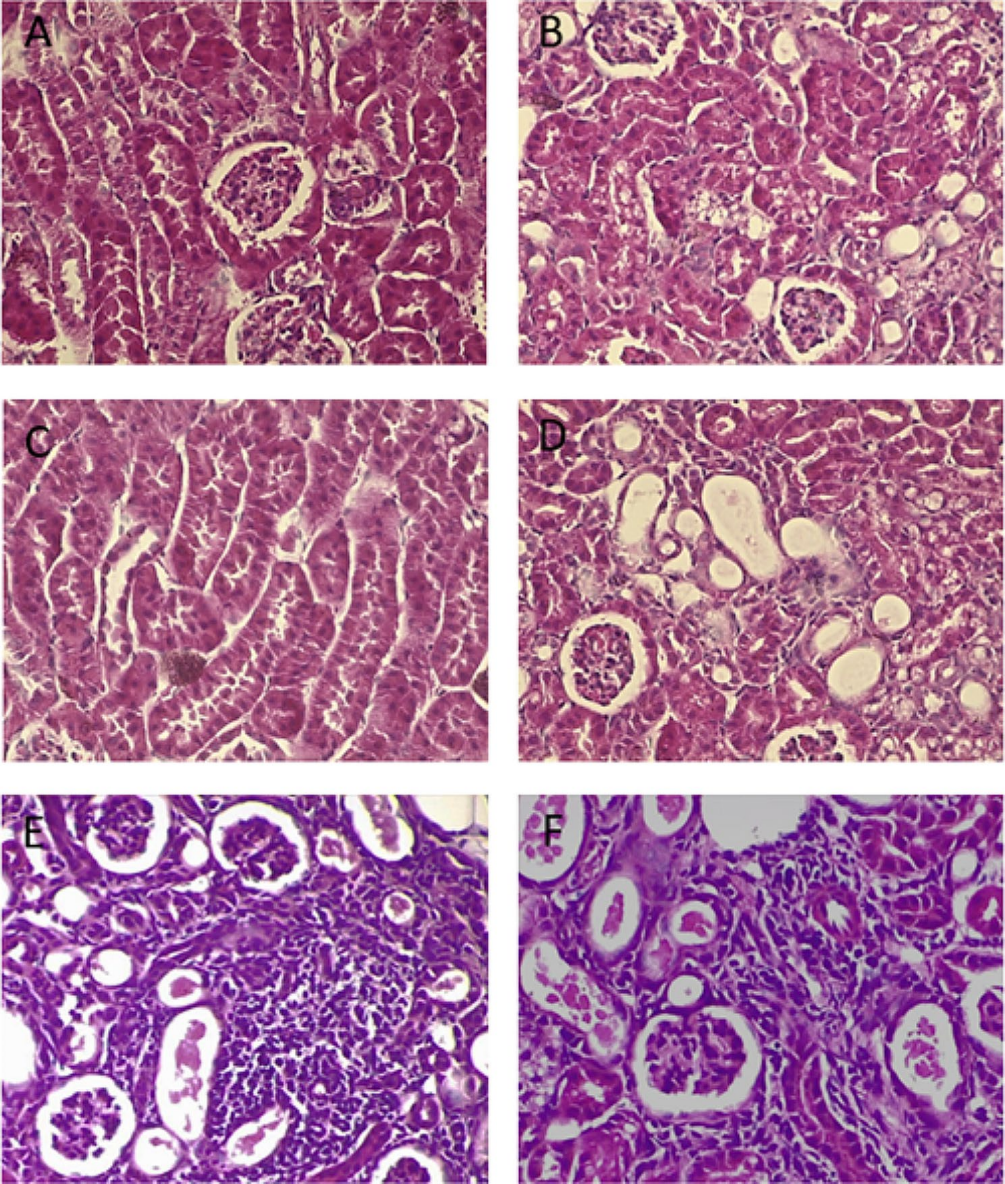
Light microscopy images showing pathological lesions found in acute tubular necrosis (Panels **A-D**) and acute interstitial nephritis (Panels **E-F**), 40x magnification. Panel (**A**) Hydropic degeneration of the kidney tubules. Panel (**B**) Vacuolization of the cytoplasm of the tubular epithelial cells. Panel (**C**) Cell detachment and the presence of detached cells in the lumen of the tubules. **D** Dilation of the tubules. **E** and **F**) Leukocytes infiltrating the interstitial area

Figure 4 shows illustrative light microscopy images of the lesions found in the study.

### Treatment with cisplatin plus anti-PD-L1 antibodies induces strong PD-1 glomerular staining in mice but not in humans

**Fig. 5:**
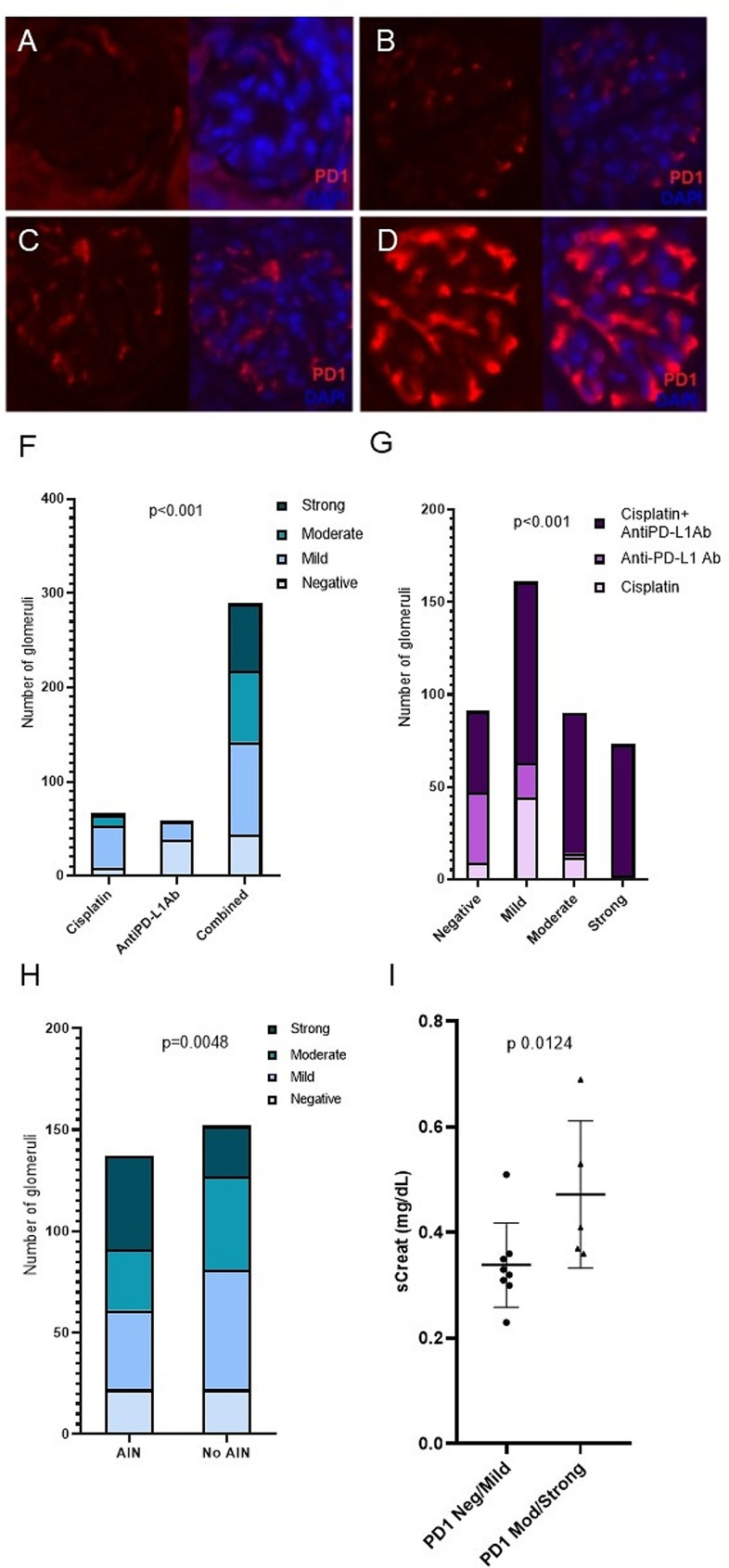
PD-1 glomerular immunofluorescence staining (red) was evaluated at 20x and semiquantitatively scored into 4 categories: absent (panel **A**), mild (panel **B**), moderate (panel **C**), and strong (panel **D**). Nuclei were counterstained with DAPI (blue). Panel F shows the distribution of the grade of PD-1 staining across the different treatments received by mice. Panel G shows the distribution of the different treatments administered to mice according to the grade of PD-1 glomerular staining. Panel H shows the proportion of the different grades of PD-1 staining in mice treated with cisplatin plus anti-PD-L1 antibodies depending on the presence of AIN. Panel I shows the mean serum creatinine found in mice with predominance of PD-1 negative or mild staining compared to those with predominantly moderate or strong staining

We evaluated the intensity of glomerular PD-1 staining in mice using a semi quantitative scale (Fig. 5, Panel A-D). Mice receiving cisplatin plus anti-PD-L1 antibodies showed a higher proportion of glomeruli with strong PD-1 positivity compared to the other groups (*p* < 0.001) (Fig. 5, Panel F). Among the total glomeruli evaluated, the highest proportion showing strong PD-1 immunostaining corresponded to animals receiving cisplatin plus anti-PD-L1 (*p* < 0.001) (Fig. 5, Panel G). Among mice receiving cisplatin plus anti-PD-L1, those with AIN showed a higher proportion of glomeruli with positive PD-1 staining (*p* = 0.0048) (Fig. 5, Panel H). Serum creatinine was higher in mice with glomerular PD-1 staining predominantly moderate or strong compared with mice with predominantly mild or negative staining (*p* = 0.0124). Interstitial infiltrates in mice who developed AIN showed positive PD-1 staining.

**Fig. 6:**
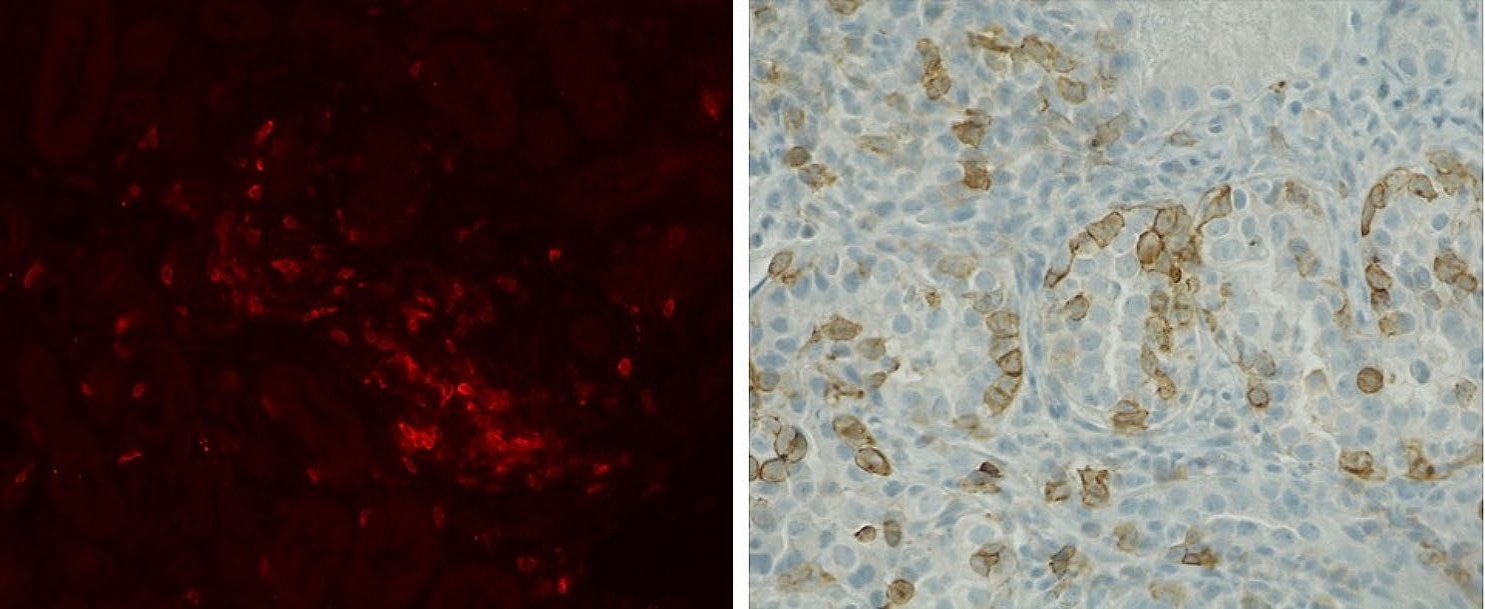
The interstitial infiltrates in mice AIN are PD-1 positive (Panel **A**), as well as the interstitial infiltrates observed in human AIN (Panel **B**)

We also evaluated PD-1 glomerular staining in 14 kidney biopsies from patients diagnosed with AIN. Of those, 10 patients were treated with ICI, and 5 received treatment with platinum derivatives. None of the evaluated kidney biopsies showed positive glomerular PD-1 staining. In line with the finding in mice, interstitial infiltrates in patients diagnosed with AIN showed PD-1 positivity. (Fig. 6).

### Inflammation-related cytokines in AIN induced by cisplatin plus anti-PD-L1 antibodies in mice

Serum and/or urine samples were available from twenty mice. Urinary MCP1 (uMCP1) was higher in mice with AIN compared to mice who did not develop AIN (Table 2). Moreover, uMCP1 correlated to serum creatinine (r 0.52 *p* value 0.02).

**Table 2:** Serum and urinary concentration of the studied cytokines in mice with and without acute interstitial nephritis (Interleukin-6 (IL-6), interferon gamma (IFNγ)-induced protein 10 (IP-10)/C-X-C motif chemokine ligand (CXCL)-10, tumor necrosis factor (TNF)α, and monocyte chemoattractant protein-1 (MCP1))

	AIN (*n* = 6)	no AIN (*n* = 14)	*p* value
sTNFα	5.48 ± 6,31	2,96 ± 3,75	0,46
sCXCL10	120,4 ± 107,0	91,92 ± 121,3	0,4
sIL6	6,84 ± 5,88	8,27 ± 8,73	0,94
sMCP1	11,46 ± 6,85	8,51 ± 3,11	0,16
uTNFα	1,58 ± 0,80	2,33 ± 2,13	0,5
uCXCL10	145,5 ± 183,4	93,28 ± 49,24	0,19
uIL6	6,95 ± 3,61	12,69 ± 17,32	0,19
uMCP1	8,35 ± 8,15	5,34 ± 1,29	**0,013

We compared the serum and urinary concentration of the cytokines in the different treatment groups. Serum IL-6 was higher in animals treated with cisplatin plus anti PD-L1 antibodies compared to those treated with the two drugs in monotherapy, but the differences only were statistically significant when compared with cisplatin alone (p 0.008). There were no differences in serum levels of TNFα, CXCL10 and MCP1 nor in the urinary levels of any of the cytokines among the different treatment groups. (see supplementary Fig. 2).

### uMCP1 in AIN related to ICI and ATN related to platin derivatives in cancer patients

**Fig. 7:**
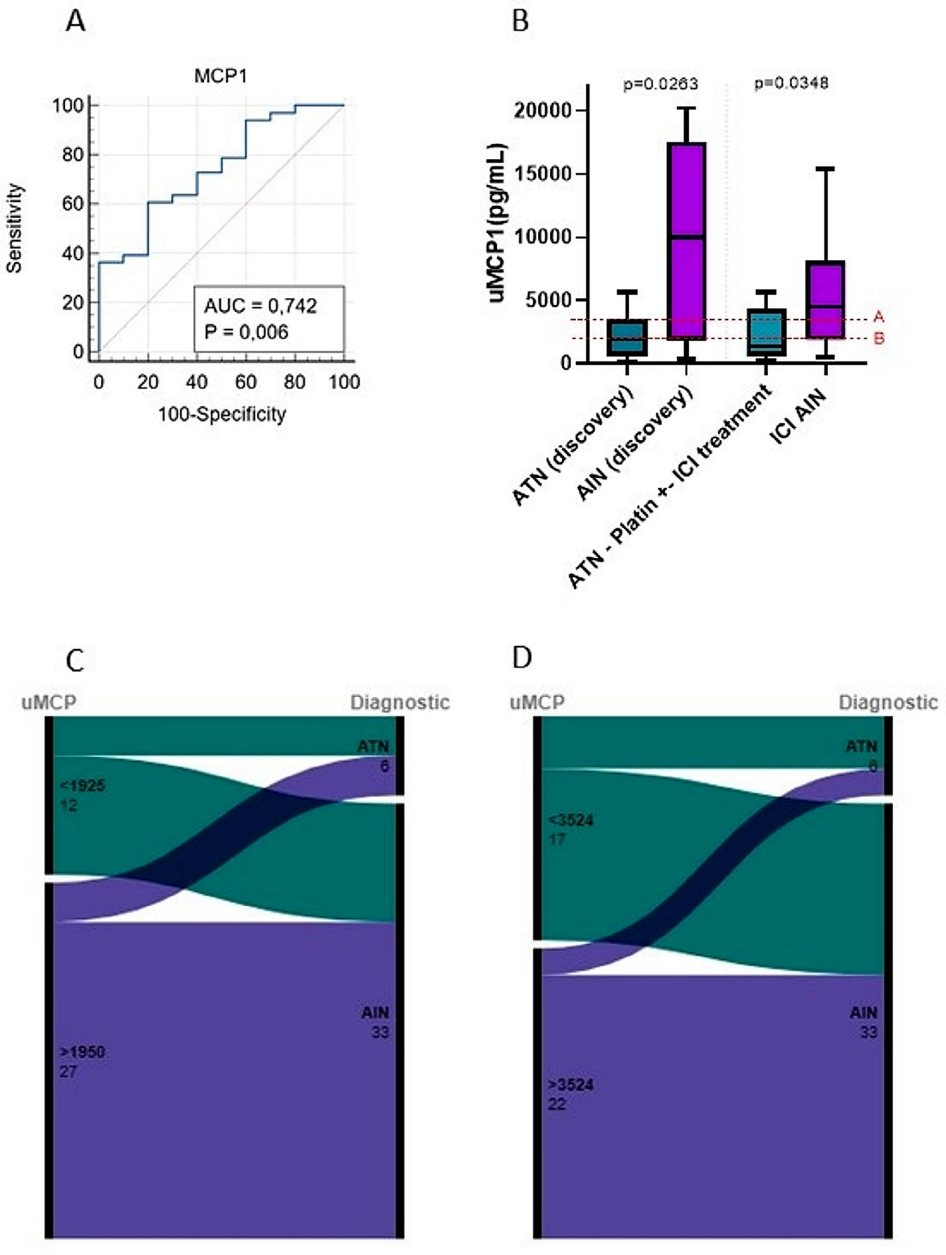
Evaluation of uMCP as a biomarker of AIN related to ICI in cancer patients. Panel **A** shows the ROC curve for the discriminative performance of uMCP between ICI-related AIN and ATN in cancer patients. Panel **B** shows uMCP levels in AIN compared to ATN in the discovery and validation cohorts. Cutoff A corresponds to the 75th percentile of uMCP in patients with ATN in the discovery cohort, whereas cutoff B corresponds to the 50th percentile in the same group. Panels **C** and **D** show alluvial diagrams illustrating the distribution of patients according to the two established cutoffs and the confirmed diagnosis

We have previously described higher levels of uMCP1 in a cohort of patients suffering AIN from different causes when compared to ATN (henceforth, *discovery cohort*) [16]. Now, we are aiming to validate these findings in a cohort of cancer patients treated with ICI and platinum derivatives, and diagnosed with AIN or ATN (henceforth, *validation cohort*). Baseline characteristics of the validation cohort are shown in Table 3. In line with our previous study, in the validation cohort, uMCP was higher in patients diagnosed with ICI-AIN compared to patients diagnosed with ATN (4474 pg/mL (IQR 1874-8092pg/mL) in ICI-AIN vs. 1407 pg/mL (IQR 4030 − 591.2 pg/mL) in ATN, p 0.0348). A ROC curve illustrating the classification performance of uMCP1 in distinguishing between ATN and ICI-AIN was plotted (Fig. 7, Panel A). In this ROC curve, AUC was 0.773 (p 0.015)).

**Table 3:** Baseline characteristics of the validation cohort

	ATN (*n* = 6)	AIN (*n* = 33)	*P* value
Age (years)	64.89 ± 5.36	68.06 ± 12.39	0.317
Blood eosinophil count (cellsx10^6^/L)	226.66 ± 136.18	280 ± 378	0.738
CRP (mg/L)	9.77 ± 10.28	75.86 ± 102.10	0.049
Urinary protein to creatinine ratio (g/mol)	23.55 ± 19.34	30.34 ± 19.46	0.226
Urine leukocyte count (cells/µL)	16.17 ± 10.98	49.35 ± 65.10	0.226
Hypertension (% patients)	66.7	42.4	0.405
Leucocyturia (%patients)	66.7	81.8	0.245
Hematuria (% patients)	16.7	9.1	0.52
Treatment with ICI (% patients)	100	100	0.99
Treatment with platinum derivatives (% patients)	100	42.4	< 0.001
Urine erytrocyte count (cells/µL)	2.83 ± 3.25	10.67 ± 40.41	0.641
sCreatinine (µmol/L)	180 ± 58.79	231.13 ± 103.22	0.251
Previous CKD (% patients)	16.6	6.1	0.522

In addition, two cutoff values of uMCP1, corresponding to the 50th and 75th percentiles of uMCP1 in the ATN group of the validation cohort, were established (Fig. 7, Panel B). Sensitivity, specificity, and predictive values were then calculated for these two cutoffs in the distinction between ICI-AIN and ATN (see Table 4). Alluvial diagrams, illustrating the distribution of patients from the validation cohort based on the uMCP1 cutoff values and confirmed diagnoses, are presented in Fig. 7, **Panels C and** D.

**Table 4:** Sensitivity, specificity, and predictive values calculated for both the discovery and validation cohorts according to two pre-stablished cutoffs based on the discovery cohort. The aim was to distinguish between AIN and ATN. Cutoff A corresponds to the 75th percentile of uMCP in patients with ATN from the discovery cohort, whereas cutoff B corresponds to the 50th percentile of uMCP in the same group. AIN-Acute interstitial nephritis; ATN – acute tubular necrosis

Cohort	Cutoff value (pg/mL)	Sensitivity (%)	Specificity (%)	PPV (%)	NPV (%)	PLR
Discovery	> 3524 (A)	64,71	80	84,64	57,11	3,23
	> 1925 (B)	76,47	50	72,26	55,51	1,53
Validation	> 3524 (A)	60,62	80	90,90	38,11	1,41
	> 1925 (B)	72,73	50	82,75	35,72	1,455

**Fig. 8:**
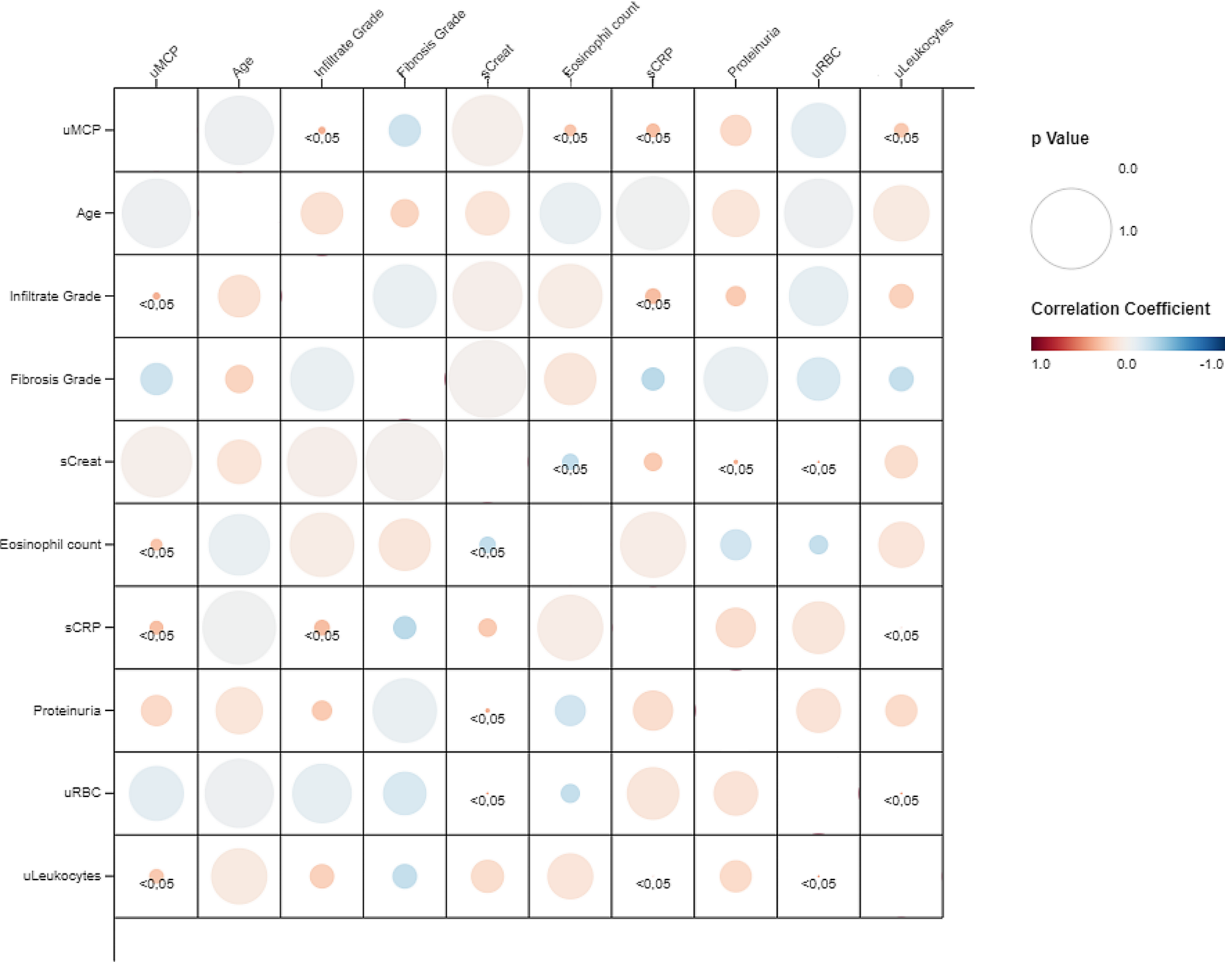
Fig. 8 shows a correlation matrix of the clinical variables evaluated and urinary MCP1

In the validation cohort, uMCP1 correlated significantly to peripheral blood eosinophil count, the urinary leukocyte count and serum C-reactive protein. Moreover, uMCP1 correlated to the grade of the interstitial infiltrate in the kidney biopsy (see Fig. 8).

## Discussion

Over recent years, the combination of cisplatin plus ICI has been established as the first line treatment of some neoplasms due to the synergy between those two compounds in reducing the intratumoral immunosuppressive milieu. This synergy increases the percentage of patients who can benefit from immune checkpoint blockade therapy. Nevertheless, the nephrotoxicity of the combined treatment – particularly with an appreciable incidence of AIN - is higher compared to the nephrotoxicity of the drugs in monotherapy. The rodent model of cisplatin acute kidney injury has been extensively described, but to the date there is a lack of animal models of ICI associated AKI. Moreover, few authors have examined the additive nephrotoxicity of the combination with cisplatin.

As expected, based on clinical reports from patients treated with these therapies, our results show that serum creatinine was significantly higher in mice treated with cisplatin plus anti PD-L1 antibodies compared to the drugs in monotherapy. The rise in serum creatinine occurred irrespective of the schedule of treatment administration but it was more severe when the drugs were administered simultaneously the same day. This finding has not been described in previous literature and may suggest that the administration of cisplatin and checkpoint blockade therapy should be better if it were scheduled on different days, thus enhancing it as a strategy to reduce nephrotoxicity.

In contrast to the study published by Spielbauer et al [11], who did not find structural lesions, we identified extensive damage in the most affected mice, which might be associated with the renal failure. 95% of individuals in the combined treatment group presented with ATN lesions and 40% of the mice in this group presented AIN. Moreover, ATN lesions were more severe in mice who were receiving the combined treatment compared to those receiving only cisplatin. None of themice treated with the drugs in monotherapy presented AIN. Spielbauer et al. hypothesized that there being no renal lesions in their study may be due to a delay of 3 weeks between the last treatment and the euthanasia of the mice. Instead, we euthanized mice 7 days after the drug administration, and thus were able to observe these renal lesions in an earlier stage.

Additionally, the higher serum creatinine level observed in mice with AIN compared to that in mice without AIN, together with the positive correlation of the number of areas of AIN with the serum creatinine, indicated that the presence of these inflammatory lesions is clinically relevant.

We detected PD-1 expression by immunofluorescence in the interstitial infiltrates in mice with AIN, as well as in human ICI-AIN, consistent with the findings of Cassol et al [2]. PD-1 staining was also positive in glomeruli from mice, especially in those animals treated with cisplatin plus anti-PD-L1 and those who developed nephritis. Recently, Pippin et al. have described higher expression of PD-1 in glomerular podocytes from 21-month-old mice compared to young mice, suggesting glomerular PD-1 as a senescence marker. In our study, all mice were young (8–10 weeks old), leading us to hypothesize that PD-1 overexpression is due to nephrotoxicity rather than age-related factors. In line with the results from Pippin et al., we observed higher serum creatinine in mice with a higher expression of glomerular PD-1 [17].

In contrast, we did not find PD-1 staining in glomeruli from patients with ICI-related AIN, suggesting that there is lower glomerular involvement in ICI and platinum derivatives’ renal damage in humans compared to that in mice. In fact, PD-1 knockout mice develop a lupus-like glomerular disease whereas glomerular involvement is infrequent in patients with renal ICI-associated irAEs [18].

Our group previously published a study comparing the urinary concentration of a panel of ten cytokines in a cohort of patients diagnosed with AIN and a cohort of patients with ATN, yielding promising results in terms of IL6, TNFα, CXCL10, and MCP1 as AIN urinary biomarkers. In line with these findings, we observed higher levels of three inflammatory cytokines—TNFα, CXCL10, and MCP1—in both serum and urine. Statistical significance was only reached with urinary MCP1.

Subsequently, we tested the biomarker performance of urinary MCP1 in a cohort of cancer patients treated with ICI and/or platinum derivatives to distinguish between ICI-AIN and ATN. As shown, we validated the increase in uMCP1 from our previous study in patients with AIN, and how it is parallel with the results in the present animal model of AIN. Altogether, the findings from our clinical and preclinical studies suggest the role of MCP1 in AIN and the utility of this molecule as AIN biomarker. High urinary MCP1 levels have been described in other immune mediated renal diseases such as lupus nephritis [19] and antineutrophil cytoplasm antibodies (ANCA) associated vasculitis [20]. MCP1 has a key role in inflammatory processes as chemoattractant of immune cells and enhancer of the expression of other pro-inflammatory factors [21].

The main strength of our study lies in the potential of the proposed animal model as a tool to enhance research on the pathophysiology of the disease, identify non-invasive biomarkers, and assess novel strategies, both for preventing its occurrence and effectively treating it after it has occurred. As potential confounders, mice in our study were young and had not any neoplasm, thus the potential influence of age and cancer-related inflammation in the incidence of AIN have not been accounted [22].

## Conclusions

In summary, this study demonstrates the induction of AIN in a significant proportion of mice treated with a combination of cisplatin and anti-PD-L1 antibodies. AIN induced by the combination of both drugs results in kidney damage, which is more severe when the drugs are administered simultaneously, suggesting that scheduling the administration of treatments on different days can reduce nephrotoxicity. The increase in uMCP1 associated with AIN in mice is also observed in human ICI-AIN, indicating the potential utility of this molecule as both a biomarker and a suitable therapeutic target, although the latter needs to be confirmed in future studies. We also observed differences in PD-1 glomerular staining in mice with AIN compared to ICI-AIN patients, suggesting a distinct involvement of the glomerular compartment in the toxicity elicited by these drugs.

### Electronic supplementary material

Below is the link to the electronic supplementary material.

Supplementary Material 1. Supplementary figure 1 shows dipstick evaluation of the urine according to the treatment received.

Supplementary Material 2. Supplementary figure 2 shows the serum and urine level of the inflammatory cytokines according to the treatment received.

## Data Availability

The datasets used and/or analyzed during the current study are available from the corresponding author(s) on reasonable request.

## Abbreviations

ICI: Immune checkpoint inhibitors
PD-1: Programmed cell death protein 1
PD-L1: Programmed cell death ligand 1
PD-L2: Programmed cell death ligand 2
irAEs: Immune-related adverse effects
AIN: Acute interstitial nephritis
AKI: Acute kidney injury
IL: interleukin
TNFα: Tumor necrosis factor alpha
ATN: Acute tubular necrosis
IP: Intraperitoneally
RT: Room temperature
IFNγ: Interferon gamma
CXCL: C-X-C motif chemokine ligand
uMCP1: Urine MCP-1
