# Supplementary figures and images for: Immune checkpoint inhibitors induce acute interstitial nephritis in mice with increased urinary MCP1 and PD-1 glomerular expression
Source: J Transl Med. 2024 May 3;22:421. doi: 10.1186/s12967-024-05177-9 (PMC11069287; doi:10.1186/s12967-024-05177-9)

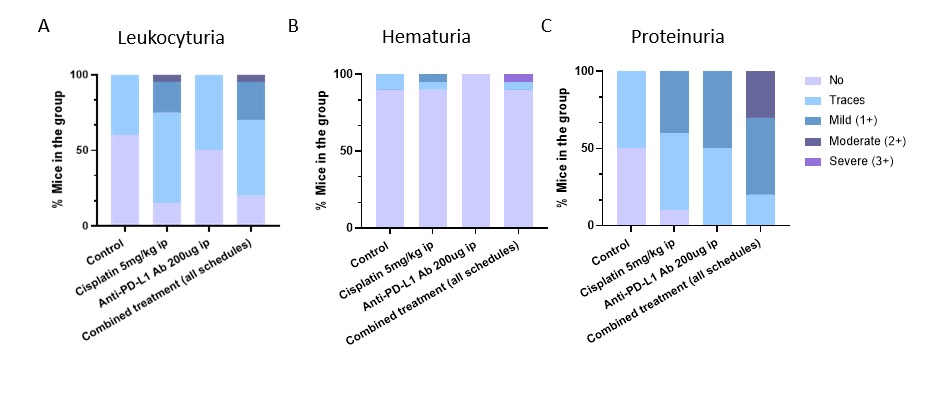

Supplement: Supplementary file 1 — Supplementary Material 1. Supplementary figure 1 shows dipstick evaluation of the urine according to the treatment received. [file 12967_2024_5177_MOESM2_ESM.jpg]

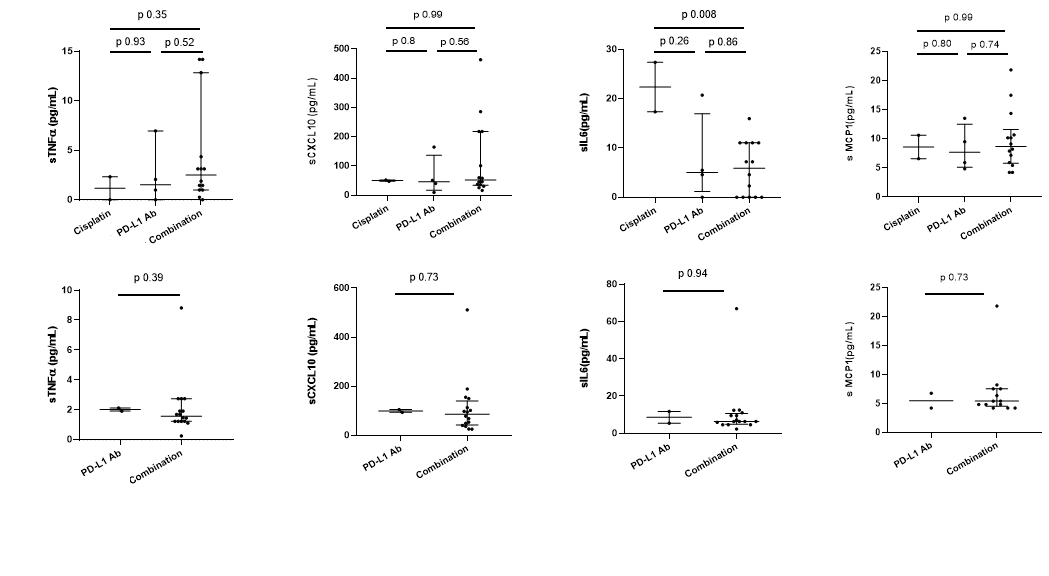

Supplement: Supplementary file 2 — Supplementary Material 2. Supplementary figure 2 shows the serum and urine level of the inflammatory cytokines according to the treatment received. [file 12967_2024_5177_MOESM1_ESM.tif]
